# Acceptive Immunity: The Role of Fucosylated Glycans in Human Host–Microbiome Interactions

**DOI:** 10.3390/ijms22083854

**Published:** 2021-04-08

**Authors:** Svetlana Kononova, Ekaterina Litvinova, Timur Vakhitov, Maria Skalinskaya, Stanislav Sitkin

**Affiliations:** 1 Department of Microbiology, State Research Institute of Highly Pure Biopreparations, 197110 St. Petersburg, Russia; tim-vakhitov@yandex.ru (T.V.); mskalinskaya@yahoo.com (M.S.); drsitkin@gmail.com (S.S.); 2 Institute of Protein Research, Russian Academy of Sciences, 142290 Pushchino, Russia; 3 Scientific-Research Institute of Neurosciences and Medicine, 630117 Novosibirsk, Russia; litvinovaea@physiol.ru; 4 Siberian Federal Scientific Center of Agro-BioTechnologies, Russian Academy of Sciences, Krasnoobsk, 633501 Novosibirsk, Russia; 5 Department of Internal Diseases, Gastroenterology and Dietetics, North-Western State Medical University Named after I.I. Mechnikov, 191015 St. Petersburg, Russia; 6 Institute of Perinatology and Pediatrics, Almazov National Medical Research Centre, 197341 St. Petersburg, Russia

**Keywords:** intestinal microbiota, acceptive immunity, immune tolerance, fucose, fucosylated glycans, host–microbiome interactions

## Abstract

The growth in the number of chronic non-communicable diseases in the second half of the past century and in the first two decades of the new century is largely due to the disruption of the relationship between the human body and its symbiotic microbiota, and not pathogens. The interaction of the human immune system with symbionts is not accompanied by inflammation, but is a physiological norm. This is achieved via microbiota control by the immune system through a complex balance of pro-inflammatory and suppressive responses, and only a disturbance of this balance can trigger pathophysiological mechanisms. This review discusses the establishment of homeostatic relationships during immune system development and intestinal bacterial colonization through the interaction of milk glycans, mucins, and secretory immunoglobulins. In particular, the role of fucose and fucosylated glycans in the mechanism of interactions between host epithelial and immune cells is discussed.

## 1. Introduction

The second half of the past century and the first two decades of the new century are characterized by an increase in the number of chronic non-communicable diseases, such as cardiovascular diseases, type 2 diabetes, obesity, chronic liver disease, inflammatory bowel disease (IBD), allergic and autoimmune diseases, and various cancers. Studies of recent decades show that this is largely due to a disruption of the relationship between the human body and its symbiotic microbiota, and not pathogens. The human body and its microbiota form a single ecological system—a superorganism [1]. The human gastrointestinal (GI) tract is an ecological niche for 10^13^–10^14^ bacterial cells, many of which are in a mutualistic relationship with the host. Over a thousand bacterial species have been found in the human gut, of which at least 160 species are present in every human [2]. Not only are they involved in nutrient absorption or production, affecting energy metabolism and determining the host metabolic phenotype, they also play a critical role in developing and maintaining immune and intestinal barrier functions [3,4]. The relationship with symbionts differs from that with pathogens: it is not accompanied by the development of inflammation but is a physiological norm. This is achieved due to the presence of trophic and regulatory links between the microbiota and the macroorganism, and by the control of the microbiota by the immune system; only a disturbance of this balance can trigger pathological mechanisms. The intestinal immune system experiences the greatest antigenic load in the human body, constantly facing a huge amount of microbial and dietary antigens. This should not only induce tolerance, but also maintain the ability to respond to various dangerous challenges.

Consideration of the immune system from this viewpoint led to V.B. Klimovich proposing the concept of functional immunity, which combines the innate and adaptive immunity mechanisms. According to this concept, immunity can be divided into: (1) protective (protecting against pathogens), and (2) acceptive (responsible for homeostatic relationships with symbiotic microorganisms) [5]. Acceptive immunity includes such mechanisms as: (1) the recognition of conserved motifs of molecular structures—the microbial patterns of microorganisms—by pattern recognition receptors; (2) the production of mucus, including secretory IgA (sIgA) and mucins, and antibacterial peptides by the host barrier tissues; (3) the induction of T helper (Th) Th17, Th1, Th2, and regulatory T (Treg) cells and anti- and pro-inflammatory cytokines.

Recently, Byndloss et al. proposed a microbiota-nourishing immunity hypothesis [6], where they assigned a key role of preserving anaerobiosis in the gut lumen in support of a healthy microbiome. However, the control of healthy microbiota formation by the host also undergoes pattern discrimination by the immune system and ensures access to host glycans. Epithelial and immune cells primarily come into contact with carbohydrate structures exposed on the bacterial cell surface, which are the main candidates for the role of the primary markers for such selection. Various prokaryotes can express both limited and wide numbers of glycoforms based on different monosaccharides, including unusual monosaccharides, which can be further enzymatically modified by different chemical groups. The composition of mammalian *N*- and *O*-glycans is limited to 10 monosaccharide units that can also be modified [7]. However, such monosaccharides as L-fucose (Fuc), mannose (Man), galactose (Gal), and some sialic acids are often found in both prokaryotes and eukaryotes, and can form similar carbohydrate patterns [8,9]. The synthesis of carbohydrate patterns similar to that of the host by microbial commensals and helminths is a molecular mimicry that allows them to achieve recognition by the host’s immune system during their colonization.

Fuc-containing patterns are involved in many cellular processes, including the mechanisms of switching the immune response [10], and fucosylated host glycans play an important role in intestinal microbiota formation [11]. We will discuss the role of bacterial Fuc-containing patterns in the formation of the acceptive immune response to symbiotic microbiota.

## 2. Recognition of Bacterial Surface Antigens

During colonization, the microbiota interacts with the immune system through the production of metabolites and the presentation of different patterns to immune and epithelial cells at different sites in the body [12].

### 2.1. Toll-Like Receptors (TLR)

The receptors TLR2, TLR1, TLR6, and TLR4 recognize bacterial cell wall components; TLR5 recognizes flagellin. They are expressed by both immune cells (macrophages and dendritic cells [DCs]) and intestinal epithelial cells (IECs): TLR2 and TLR4 are mainly in the small intestine, while TLR5 is in the human colon [13].

TLR4 is the lipopolysaccharide (LPS) receptor of gram-negative bacteria that forms homodimers. TLR2 can form homodimers and heterodimers with TLR1 or TLR6. The cluster of differentiation 14 (CD14) protein broadens the spectrum of molecular structures of both gram-positive and gram-negative bacteria and yeast recognized by the two. Heterodimer TLR2/1 senses triacyl lipoproteins found mainly in gram-negative bacteria, thermolabile enterotoxins, yeast lipomannan/lipoarabinomannan, and porins. TLR-2/6 recognizes diacyl lipoproteins and lipoteichoic acid of gram-positive bacteria [13].

TLRs use two sets of functional adapter proteins for sorting and signaling: (1) toll–interleukin 1 receptor (TIR) domain–containing adaptor protein (TIRAP) and myeloid differentiation primary response 88 (MYD88), and (2) TIR domain-containing adapter-inducing interferon (IFN) beta (TRIF) and TRIF-related adaptor molecule (TRAM), which provide signal transmission from the cell plasmalemma and endosomes, respectively [14].

All TLRs use the MYD88-dependent pathway, where the signal activates the nuclear factor kappa light chain enhancer of activated B cells (NF-κB) and protein kinases: c-Jun N-terminal kinase (JNK) and p38 mitogen-activated protein kinase (MAPK p38) [15]. Transforming growth factor beta (TGF-β) can interfere with TLR2, TLR4, and TLR5 ligand-induced responses and contribute to MYD88 ubiquitination and degradation [16]. Interleukin (IL)-10 also induces the ubiquitination and subsequent protein degradation of MYD88-dependent signaling molecules [17]. Although TLR2 activation in this pathway mainly leads to the induction of pro-inflammatory cytokines, TLR2 heterodimers formation allows immune response modulation: TLR2/6 heterodimers induce a pro-inflammatory response, while TLR2/1 heterodimers can induce an anti-inflammatory response through IL-10 expression based on the ligand [13].

TLR4 can signal through either the MYD88-dependent or MYD88-independent pathways. The MYD88-independent pathway is associated with IFN-β stimulation and DC maturation. The initiation of type I IFN transcription occurs after the phosphorylation of IFN regulatory factor 3 (IRF3) and its translocation into the nucleus in a complex with other proteins [14]. TLR4 requires CD14 in completing TRIF to regulate IRF3 [13]. TLR4 or TLR2/6 signaling through the MYD88-dependent pathway occurs in the case of smooth LPS with abundant *O*-glycated lipid A, but in the case of rough LPS or lipid A detection, TLR4 and CD14 form a complex with TRIF [15]. TLR4 and TLR2 also bind to myeloid differentiation factor 2 (MD-2); however, MD-2 binding to TLR2 is weaker than its binding to TLR4. The presence of MD-2 allows TLR4 to respond to wide range of endotoxic partial structures of gram-negative bacteria LPS, gram-negative bacteria, and lipoteichoic acid of gram-positive bacteria but not to gram-positive bacteria, peptidoglycan or lipopeptide. MD-2 allows TLR2 to respond to protein-free endotoxic LPS, deep-rough chemotype LPS (ReLPS), and lipid A, and enhances TLR2-mediated responses to gram-negative and gram-positive bacteria, protein-containing LPS, peptidoglycan, and lipoteichoic acid. MD-2, TLR2, and TLR4 mutually enhance each other’s expression in human monocytes [18]. The human milk oligosaccharide (HMO) lacto-*N*-fucopentaose (LNFP) III interacts with the TLR4–MD-2–CD14 complex of mouse bone marrow-derived DCs through α(1,3)-Fuc, and not its lacto-*N*-neotetraose (LNnT) portion. This activates extracellular signal–regulated kinase (ERK) and NF-κB and leads to promotion of the Th2 response [19].

TLR1, TLR2, TLR4, and TLR6 also exist in soluble form (sTLRs) to prevent direct interactions between cellular TLRs and their microbial ligands, reduce immune system stimulation, and prevent acute reactions to infection. sTLR2 is found in human breast milk, amniotic fluid, and saliva, and is also produced by ECs. In milk and plasma, it forms a heterodimer with sCD14. The sTLR4 heterodimer with CD14, in addition to LPS, can recognize additional pathogenic structures [13].

### 2.2. DC-SIGN

Immune cells have a large number of proteins with C-type lectin-like domains (CTLDs) that can recognize carbohydrate patterns. Dectin-1, Man receptor, and DC-specific intercellular adhesion molecule 3 (ICAM3)-grabbing non-integrin (DC-SIGN) [20] can recognize Fuc in carbohydrate patterns, but their specificity differs. Dectin-1 is better known as a receptor for yeast β-glucans, zymosan, and laminarin [20], but it can recognize the core 1,6-Fuc on immunoglobulin G (IgG) in vitro [21]. The Man receptor has two separate C- and R-type lectin motifs: the first binds terminal Fuc, Man, or *N*-acetyl-D-glucosamine (GlcNAc); the second binds glycans with 3- or 4-*O*-sulfated *N*-acetylgalactosamine (GalNAc), or 3-*O*-sulfated Lewis x (Le^x^)/Le^a^ [22]. There are few studies on the involvement of the Man receptor and dectin-1 in immune responses upon binding to Fuc-containing patterns.

DC-SIGN is a type II transmembrane protein. It consists of an extracellular domain containing CTLDs and a hinge region, a transmembrane region, and a cytoplasmic signaling region [20,23]. The hinge region is pH-sensitive and regulates the equilibrium between monomeric and tetrameric DC-SIGN states. Upon oligomerization, the CTLD location in the tetramer confers a higher specific affinity of DC-SIGN for the ligand and enhances its binding [24]. The change in pH when this complex enters the endosomes of cells leads to the decomposition of the tetramer, the weakening of binding, and the release of the ligand [23].

DC-SIGN is expressed by immature DCs in the human peripheral tissues. It is present in a limited amount on mature or activated DCs in the lymph nodes, tonsils, and spleen, and is absent from follicular DCs or Langerhans resident DCs [23]. In the human fetus, it is expressed by fetal tissue and lung macrophages, and immature DCs [25]. Its expression on GI tract ECs is promoted by hypoxia, hemorrhagic hypotension, chronic inflammatory bowel disease, ulcers, and sepsis [26].

**Figure 1:**
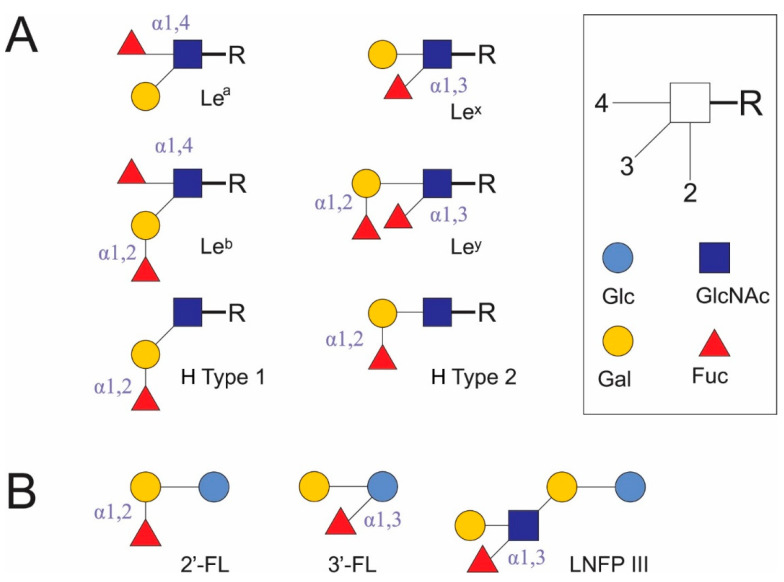
Fucosylated glycoconjugates (**A**) and some human milk oligosaccharides (**B**), which may act as unique carbohydrate patterns. Fuc: L-fucose. Gal: galactose. Glc: glucose. GlcNAc: *N*-acetyl-D-glucosamine. Le^a^: Lewis a antigen. Le^b^: Lewis b antigen. Le^x^: Lewis x antigen. Le^y^: Lewis y antigen. H type 1: H antigen type 1. H type 2: H antigen type 2. 2′-FL: 2′-fucosyllactose. 3′-FL: 3′-fucosyllactose. LNFP III: lacto-*N*-fucopentaose III.

DC-SIGN recognizes Man- and Fuc-containing antigens of several viral (human immunodeficiency virus 1, hepatitis C virus, cytomegalovirus, herpes simplex virus, influenza A virus subtype H5N1, dengue virus, Ebola virus, coronaviruses, West Nile virus, measles virus) and bacterial pathogens (*Helicobacter pylori*, *Mycobacterium tuberculosis*, *Leptospira interrogans*), fungi (*Candida albicans*, *Aspergillus fumigatus*), parasites (*Leishmania*, *Schistosoma mansoni*) [23], and commensal bacteria (*Lactobacillus rhamnosus* [27], *Bifidobacterium infantis* [28]). It also binds autoantigens, for example, the Gal-β (1,4)-[Fuc-α (1,3)]-GlcNAc trisaccharide is known as Le^x^ antigen. Le^x^ antigen has been found on crypt cells [29], as stage-specific antigen 1 (SSEA-1) [30], and a CD15 marker [31], in milk oligosaccharide LNFP III [19] (Figure 1), semen clusterin [32], and in ICAM3 on T lymphocytes [23] in mammalians. Here, it acts as a self-associated molecular pattern (SAMP) in autoantigens. At the same time, it is found in the soluble egg antigen (SEA) of helminths (*S. mansoni* [33]), *H. pylori* LPS [34], and possibly in the polysaccharides of intestinal *Bacteroides* spp. [35] and other human commensal microorganisms. In these cases, Le^x^ antigen is a pattern associated with resident microorganisms (microbe-associated molecular pattern [MAMP]) rather than pathogens (pathogen-associated molecular pattern [PAMP]) [20].

DC-SIGN preferentially binds Man or Fuc through different binding sites in the Ca^2+^-binding pocket, while GlcNAc and glucose (Glc) are less preferred. Its CTLDs contain the Glu-Pro-Asn tripeptide motif, but can also interact with terminal Gal despite the absence of a Gln-Pro-Asp motif. DC-SIGN shows greater avidity for oligosaccharides compared to monosaccharides [36].

DC-SIGN binds different Man-containing structures, including high-Man *N*-glycans [23], when interacting with oligomannose oligosaccharides; its Ca^2+^ sites are more likely to be involved in binding internal sugars than terminal sugars [37]. Human DC-SIGN and mouse homologues of DC-SIGN or closely related human DC-SIGN-related (DC-SIGNR, or L-SIGN) receptors (mDC-SIGN, mSIGNR1–8) recognize the Fuc of terminal Le^x^, Le^y^, and Le^a^, and Le^b^ glycan structures [38] (Figure 1). DC-SIGN and mSIGNR1 bind Fuc in bovine serum albumin conjugates better than Man, and bind the Le^x^ trisaccharide more avidly than Fuc [36]. Of the Le antigens in glycoconjugates, only Le^x^ and Le^y^, but not Le^a^ and Le^b^, induce a Th2 response and inhibit the LPS-induced IL-12p70 production. The addition of mannan abolishes the inhibitory effect of Le^x^ antigens [38]. As a rule, sialylated Le antigens, including sialyl-Le^x^, bind poorly to DC-SIGN. The efficiency of DC-SIGN binding decreases from Le^b^ → Le^y^ → Le^a^ → Le^x^ and LDNF (GalNAc[Fuc-αl,3]β1,4 GlcNAc-R); however, doubling the content of Le^x^ and LDNF epitopes in diantennary *N*-glycans leads to a 4-fold increase in DC-SIGN–Fc binding to them as compared to single epitopes. 1,3-Core fucosyl Man_3_ GlcNAc_2_ does not bind to DC-SIGN. Oligomerization increases the multivalence of DC-SIGN and leads to the loss of free Man and glycoconjugate binding to sialyl- or 6-sulfated Le^x^; however, binding to other glycoconjugates increases in the direction of α-L-Fuc → H antigen type 2 → Manα1,3 (Manα1,6) Man → 6-sulfated Le^a^ → Le^y^ → Le^x^ → Le^b^ [39]. Study of the binding selectivity of DC-SIGN to the HMOs 2′-fucosyllactose (2′-FL) and 3′-fucosyllactose (3′-FL) and glycans containing type 1 and 2 H antigen structures (Figure 1) has shown that they weakly bind to DC-SIGN. α1,2-Fuc in glycans appears to have a lower affinity than structures containing Le^a^ and Le^x^ [40,41].

The specificity of the immune response is achieved both through carbohydrate-specific discrimination of the DC-SIGN ligands, which triggers various signaling pathways, and through its interactions with TLR2 or TLR4, and the presence of certain cytokines. DC-SIGN binding to certain microorganisms can lead to the inhibition or stimulation of Th1 cell polarization, Th2 responses, and/or the induction of Treg cell differentiation. DC-SIGN–mediated signaling depends on TLR-induced activation of NF-κB in human monocyte-derived DCs (moDCs) [42].

The proximal signaling complex DC-SIGN (signalosome) consists of scaffold proteins LSP1, KSR1, and CNK. It is required for the constitutive recruitment of serine/threonine-specific protein kinase Raf-1 into DC-SIGN. DC-SIGN binding to Man-containing ligands results in Raf-1–dependent signaling that modulates TLR4 signaling and enhances IL-10, IL-12, and IL-6 expression. The binding to Fuc-containing ligands leads to Raf-1–independent, but LSP1-dependent, signaling due to dissociation of the KSR1–CNK–Raf-1 complex from the signalosome, which increases IL-10 expression and suppresses IL-12 and IL-6 expression [42]. For example, the ligation of Man on intracellular pathogens such as fungi, viruses, and mycobacteria with DC-SIGN together with TLR signaling leads to protective Th1 responses. Fuc-containing ligands, in particular those carrying Le^x^, induce the differentiation of Th2 and T follicular helper (Tfh) cells during cross-interactions between the DC-SIGN and TLR signaling pathways and the IFN type 1 receptor (IFNAR). The interaction of Le^x^ antigen from *H. pylori* LPS or *S. mansoni* SEA with DC-SIGN results in a Th2 immune response. The loss of Le antigen from *H. pylori* LPS triggers a Th1 immune response [43]. At the same time, Le^x^-mediated interaction between the ß(2)-integrin Mac-I on neutrophils with DC-SIGN on immature DCs in the presence of TNF-α produced by activated neutrophils leads to Th1 polarization of T cells [44]. Cross-interaction between DC-SIGN and TLR2 in DCs can induce forkhead box P3 (Foxp3^+^) Treg cell production by some strains of *L. rhamnosus* [27] and *B. infantis* [28]; however, the recognizable pattern is unknown in this case. Despite the above findings, the specific signaling mechanisms are still poorly understood [23].

Accordingly, the question of which patterns are used by bacteria to establish homeostatic relations with the host and how this happens arises.

## 3. Stages of Immune System Development in the First Year of Life

**Figure 2:**
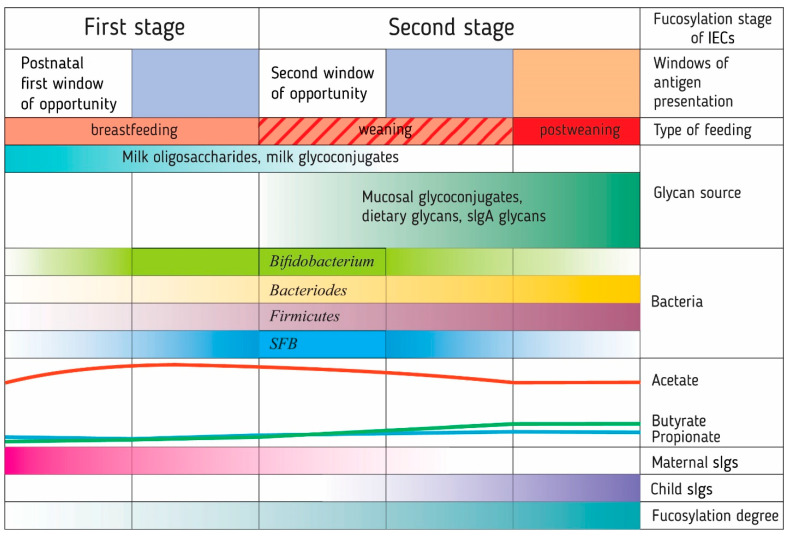
The formation of a ‘layered’ immunity in an infant through a number of sequential processes associated with fucosylation. These processes have several critical time periods, called windows of opportunity, during which the immune system is trained by antigens presented to it. The presentation of fucosylated patterns of food (oligosaccharides and glycoconjugates of breast milk) and bacterial antigens (*Bacteroides*) for the development of immune tolerance to them probably occurs before the opening of the second window of opportunity in the development of the immune system during breastfeeding. The synthesis of carbohydrate patterns in bacteria is determined by the type of glycans available for metabolism by intestinal bacteria. The introduction of solid foods in infancy opens a second window of opportunity due to the presentation of new food and bacterial antigens. Glycan-degrading *Bacteroides* stimulate the growth of Firmicutes and butyrate production, which, along with segmented filamentous bacteria (SFB), enhances the fucosylation of the intestinal epithelial glycocalyx. Human breast milk, mucin, and secretory immunoglobulin glycans are part of the host’s mechanism controlling bacterial colonization. IECs: intestinal epithelial cells. sIgs: secretory immunoglobulins.

Studies of the intestinal microbiota and immune system development in the first year of life in children have led to the formation of the neonatal window of opportunity concept and the idea of layered immunity [45,46]. They imply that the transition from the immediate postnatal period to the mature immune system of the adult organism follows a specific order of events, a series of non-redundant successive phases [45] (Figure 2).

At this time, multilevel connections within the microbiota begin to form, the innate and adaptive immune systems are initiated, and the foundation is laid for subsequent homeostatic relationships between the host and the microbiota. In particular, the development of immune tolerance to food and bacterial antigens occurs after their transport to colon lamina propria DCs via goblet cell–associated antigen passages (GAPs) [45] and through microbial metabolites. Both premature and delayed exposure to different microorganisms have far-reaching health implications and an increased risk of developing diseases at an older age, such as allergic and autoimmune diseases, obesity, IBD, and cancer [46,47,48]. This makes it important to study the mechanisms of the changes in the microbiota and the immune system during this period to understand the causes and mechanisms of non-communicable diseases.

The first phase includes the period of breastfeeding from birth to the transition to solid foods. The microbiota at this time is significantly different from that of an adult. Its composition is determined by geographic factors, the presence of siblings and pets, breast milk composition, antibiotics, gestational age, and genetic factors (the latter forms approximately 9% of the intestinal microbiota as a polygenic trait) [45]. Lack of breastfeeding, cesarean section, antibiotics use, and eliminating parasitic infections have a large negative effect on the infant’s microbiota [49,50,51].

During pregnancy, the Th1 immune response is suppressed in both the mother and the fetus to prevent overreaction to each other’s antigens. Mutual tolerance is achieved by a bias towards Th2 immunity in pregnant women, which produces a cytokine environment that similarly shifts the fetal immune response [46]. The establishment of the infant’s immune tolerance to microbial and dietary antigens requires a balance between the populations of effector CD4^+^ Th cells and Treg cells. Typically, it is established in the first weeks of an infant’s life upon contact with intestinal microorganisms. Infants born by cesarean section have delayed Th1 activation due to their altered microbial colonization [51].

During the neonatal period, the first window of opportunity is in place for establishing the tolerance of mucosal invariant natural killer T cells to subsequent environmental influences. The immune system must contact bacterial glycolipid antigens [52] to recognize a specific sphingosine base length and its charge. This determines the cytokine response and the activation of invariant natural killer T cells upon the recognition of T cell antigen receptor (TCR)-related antigens, which leads to the induction of natural killer T cell cytotoxicity in neonatal mice [53]. Moreover, TLR signaling in mouse IECs is specifically reduced during this period, which makes IECs more sensitive to TLR4 stimulation. This neonatal tolerance depends on the delivery mode. The IECs of infants born by cesarean section did not decrease the TLR signal level and were more prone to developing epithelial damage, which indicates an important role of the influence of specific types of microorganisms on the development of IEC tolerance [48]. The first dietary antigens come from mother’s milk and are in many ways similar to autoantigens. HMO mixtures interacting with DC-SIGN and TLR4 on human moDCs leads to their semi-maturation and the further generation of Treg cells from naïve CD4^+^ T cells [54]. Further, the neonatal intestinal epithelium is different from that of adults. It has almost no mature Paneth cells and no EC turnover, which leads to its closer interaction with microbiota. The protective mucus layer only begins to grow upon weaning. In adults, cell proliferation occurs at the base of the small intestinal crypts, after which the ECs leave the crypts, migrate along the crypt–villus axis, and then peel off at the villus tip [45]. In this case, undifferentiated proliferating crypt cells carry Le^x^, Le^a^, and Le^b^ antigens, while differentiated ECs carry only Le^a^ and Le^b^ antigens [29]. In infants, TLR5 expression in the absorbing enterocytes is limited to the preweaning period. *TLR5* polymorphism, leading to decreased ligand reception, reduces the risk of developing IBDs in the future, while strengthening ligand reception increases the risk of their development.

The absence of microbial colonization during the neonatal period in germ-free mice causes morphological and cellular defects in the development of the primary and secondary lymphoid organs. Germ-free mice have reduced Treg content in the colon, IgA production by B cells, IEC RegIIIγ expression, and dysregulated IL-22 expression by ILC-3 cells. However, these disorders can be repaired by microbial colonization later in life [48].

The second phase takes place during the transition from breastfeeding to solid foods, usually starting at around 6 months in infants and 2–3 weeks in mice. At this time, a change in dietary antigens begins, the support of the child’s immunity and control over the microbial composition through lactoferrin, oligosaccharides, and sIgA and sIgG of breast milk decreases, and the diversity of bacterial antigens increases significantly. It is believed that this period is the second window of opportunity in establishing oral tolerance [46].

The third phase starts after the complete cessation of breastfeeding. The microbial composition and load increase sharply, and the spectrum of dietary antigens changes. Approximation of the species and quantitative composition of the intestinal microbiota to that in adults occurs by 2–3 years in children, and by 3–4 weeks in mice [45].

## 4. Commensal Gut Microbiota and Changes in Short-Chain Fatty Acid Composition

The ability of bacteria to utilize various types of carbohydrate sources is one of the main factors determining the structure of the microbial community.

The intestinal microbiota has been described as a functionally redundant ecosystem whose stability is maintained regardless of the presence of key bacterial species [55]. However, analysis of biotic interaction structures has shown that *Bacteroides* and *Bifidobacterium* are keystone taxa [56,57]. Numerous biotic connections of *Bacteroides fragilis*, *Bacteroides stercoris*, and *Bifidobacterium* spp. define most parts of the community structure, creating locally stable conditions for other species, and modulating and stabilizing fundamental ecosystem processes. Representatives of Proteobacteria and Tenericutes, comparable in level to Actinobacteria, do not form such numerous biotic connections [57]. Bacteroidetes and *Bifidobacterium* take this position because they act as primary glycan degraders. Bacteroidetes have a large and varied glycoside hydrolase (GH) and extracellular polysaccharide lyase content, so they can degrade a wider range of glycans inaccessible to other members of the community [58]. The difference in priority degradation of different types of glycans allows them to avoid interspecies competition in an overlapping ecological niche [59]. The more glycan-specific Firmicutes are abundant in the gut as a result of cross-feeding of Bacteroidetes-degraded glycans, which Firmicutes primarily metabolize intracellularly [58]. The change in *Bifidobacterium* spp. dominance in infant intestines to the dominance of representatives of Bacteroidetes and Firmicutes in adults [49] is largely due to the change in carbohydrate sources during the transition to solid food. *Bifidobacterium* spp. also undergo a shift in species dominance. *Bifidobacterium longum* subsp. *infantis* and *Bifidobacterium bifidum*, which metabolize HMOs, are displaced by *Bifidobacterium catenulatum* and *Bifidobacterium adolescentis* [60], using the same cross-feeding strategy as Firmicutes, as they do not have polysaccharide lyases. However, among Firmicutes, some species also act as primary glycan degraders, which, apparently, also makes them key, for example, some *Ruminococcus* spp. in the human colon [61,62].

In the first year of life, due to changes in breast milk composition and new sources of carbohydrates, such as polysaccharides from solid food and bacterial exopolysaccharides (EPS), both the diversity of the GI microbiota and the type of short-chain fatty acids (SCFAs) produced and their ratio change [63,64]. This was well demonstrated in a study of the gut microbiota composition and SCFAs during the first year of life in infants from Norway and Sweden [65]. Acetate dominated during the first year of life in all age subgroups, but its abundance in feces decreased from almost 90% at 3 months to less than 70% at 12 months. Propionate was the second most common SCFA in the first 6 months of life. Its abundance doubled from 3 months to 6 months to 6.7%, and reached 11.1% by 12 months. Butyrate was first detected at 3 months, and its abundance reached almost 19% by 12 months (a 4-fold increase from 6 months to 12 months). The abundance of acetate correlated positively with Enterobacteriaceae at 12 months. Butyrate correlated positively with *Clostridium* at 6 and 12 months, and propionate correlated negatively with *Clostridium* at 12 months. Bacteroidales correlated positively with propionate at 3 and 12 months, but not at 6 months, implying that the mechanisms supporting elevated propionate levels at an early age may be related to other bacteria. Clostridiales more than doubled between 6 and 12 months, and accounted for about 70% of the intestinal microbiota by 12 months. At 12 months, two infant groups with different bacterial networks formed. This was due to the presence of different glycoside hydrolases for degrading different types of glycans, including mucins and HMOs. One bacterial network contained *Eubacterium rectale* as a major member associated with *Roseburia*, *Lachnospiraceae bacterium* NK4A136, and *Lachnospira*, and was characterized by butyrate production. All bacteria in this network correlated negatively with *Ruminococcus gnavus*, which formed another network with *Erysipelatoclostridium*, *Veillonella*, *Clostridium innocuum*, *Enterococcus*, and *Escherichia*/*Shigella*, characterized by low butyrate production. More fucosidases in *R. gnavus* than in *E. rectale* made fucosylated glycans a more accessible substrate for members of its network [65].

Even earlier introduction of complementary foods to 3-month-old infants leads to a disruption in microbial diversity and microbiota composition, and changes in SCFA production, in particular to an early increase in butyric acid concentrations [66].

SCFAs play an important role in immune tolerance development and Treg cell generation, both through their effect on DCs and by direct interaction with T cells [67]. Butyrate induces IL-10 and aldehyde dehydrogenase 1a1 expression in colonic macrophages and DCs by G protein–coupled receptor (GPR) 109A signaling in mice [68]. Therefore, it contributes to the differentiation of naïve T cells into Treg cells and suppresses the differentiation into pro-inflammatory Th17 cells. Butyrate and propionate, but not acetate, inhibit antigen-specific CD8^+^ T cell activation by suppressing pro-inflammatory IL-12 secretion from treated human DCs [69]. The proposed mechanism for the various immune effects of SCFAs is the inhibition of histone deacetylases (HDACs) [67].

The ability to metabolize host glycans (milk oligosaccharides, glycosaminoglycans, glycans of mucosal glycoconjugates), and hence the ability to adhere to them, confers some *Bifidobacterium* [70,71], *Bacteroides* [72], and Firmicutes [70,73] a competitive advantage in the intestinal ecosystem. The carbohydrate composition of the metabolized sugars in turn influences the composition of the glycans synthesized by bacteria, the cell wall glycoconjugates [74], and the carbohydrate patterns formed.

## 5. Control over the Intestinal Colonization of Infants through Breastfeeding

Besides nutrients, breast milk contains glycoconjugates and oligosaccharides, antioxidants, fatty acids, glutamine, hormones, growth factors, cytokines, sCD14 and sTLR2, and immunoglobulins for the infant. In breast milk, the high level of sIgA specific to pathogens of the mother’s GI tract helps to protect the infant from the most likely pathogens [46]. sIgA also helps maintain tissue integrity to protect against inflammatory damage [75].

In addition to the mother’s birth canal, colostrum and breast milk are sources of bacteria that colonize the infant’s GI tract [76,77]. The DNA of representatives of *Bifidobacterium*, *Lactobacillus*, *Staphylococcus*, *Streptococcus*, *Bacteroides*, *Enterococcus*, and *Clostridium* clusters IV and XIVa–XIVb have been found in breast milk, where the first four groups were dominant [78]. As milk oligosaccharides and glycoconjugate glycans are selective substrates for them [79,80,81], there is a correlation between the presence of individual HMOs and bacteria both in colostrum (Table 1) [81] and infant fecal samples [82]. The bacterial composition changes as the breast milk composition changes during lactation [76,83].

A distinctive feature of the oligosaccharide composition of human milk is the predominance of fucosylated rather than sialylated HMOs [11], determined by the activity of the maternal genes of fucosyltransferase (FUT) FUT2 (*Se* gene) and FUT3 (*Le* gene) [83,84] belonging to the host gene group affecting the gut microbiome composition [85]. In accordance with the expression of the *Se* and *Le* genes, four groups of mothers are distinguished: *Le*-positive secretors (*Le*^+^*Se*^+^), *Le*-negative secretors (*Le*^−^*Se*^+^), *Le*-positive non-secretors (*Le*^+^*Se*^−^) and *Le*-negative non-secretors (*Le*^−^*Se*^−^). Approximately 70% of women are *Le*^+^*Se*^+^ secretors, and their milk contains all types of fucosylated HMOs. About 20% of women are *Le*^+^*Se*^−^ non-secretors: their milk contains 3′-FL, LNFP II, and LNFP III, but does not contain α1,2-Fuc–containing HMOs. The milk of the remaining 9% *Le*^−^*Se*^+^ mothers contains 2′-FL, 3′-FL, LNFP I, and LNFP III, and the milk of the 1% *Le*^−^*Se*^−^ mothers does not have HMOs with α1,4-Fuc residues, but contains 3′-FL, LNFP III, and LNFP V. Occasionally, 3′-FL and LNFP III are found in non-secretors, suggesting that other FUTs are involved in their synthesis [86]. The breast milk composition in mothers delivering preterm and full-term infants also differs [83,86]. Besides, its composition is influenced by the mother’s adaptation to food and pathogens from different geographic locations [87], thereby determining the composition of the gut microbiota in children [88].

In mice, study of the influence of the *fut2* gene on gut microbiota dynamics for two generations has shown strong differences in the assembly of microbial communities over time, depending on the *fut2* genotype of the host and that of their progenitors. For the *fut2*^+/+^ grand dam, the operational taxonomic units (OTUs) *Paludibacter*, *Helicobacter*, *Oscillibacter*, and *Anaerophaga* were indicators of breeding direction, and for *fut2^−/−^*, *Meniscus*, *Tannerella*, and *Bacteroides* [89]. In humans, Ruminococcaceae (e.g., *Ruminococcus*), Lachnospiraceae, and Prevotellaceae (e.g., *Prevotella*) also differ depending on the secretory status in fecal microbial communities, although this was not statistically significant [90].

## 6. Degradation of HMOs by Microorganisms

Breast milk contains 100–200 different HMOs, which have a lactose unit (Galβ1,4Glc) at their reducing end [83]. In humans, for the first days of lactation, the colostrum and milk are dominated by HMOs with type I structure (Galβ1,3GlcNAc), which are completely metabolized by bifidobacteria, in contrast to HMOs with type II structure (Galβ1,4GlcNAc), including LNFP III and LNnT. As it favors beneficial bifidobacteria colonization, whose protective role enhances infant survival, it is believed to have conferred humans an evolutionary advantage [91]. The ability to catabolize LNnT is granted by *B. longum* subsp. *infantis*, a high advantage over *Bacteroides thetaiotaomicron*, which cannot catabolize LNnT [72].

The enzyme sequences of the metabolic degradation pathways of glycan core structures are highly similar in *Bifidobacterium* spp. and *Lactobacillus* spp. [92]. Many intestinal bifidobacteria and lactobacilli use α-1,2/α-1,3-L-fucosidases, α-2,3/α-2,8-sialidases, β-1,4-galactosidase, and endo-β-1,3-N-acetylglucosaminidase to ferment the glycans of host glycoconjugates and HMOs [70]. Almost 14% of the genes of the bifidobacterial genome are responsible for carbohydrate metabolism [60]. The different enzyme localization in bacteria determines their strategy for glycan utilizing. For example, *B. longum* subsp. *infantis* has a gene cluster encoding transport systems and intracellular glycosyl hydrolases, and *B. bifidum* exports sialidases, α-L-fucosidases, and lacto-*N*-biosidase to release lacto-*N*-biose from HMO structures, which is transferred for utilization in the cell [71,86]. Most of the bifidobacterial α-L-fucosidases belong to the CAZy [93] GH29 or GH95 families. The use of fucosylated HMOs confers a competitive advantage to several *B. longum* and *B. infantis* strains during infant breastfeeding. 2′-FL and 3′-FL from breast milk stimulate the growth of *B. longum* subsp. *infantis* (but not subsp. *longum*), *B. longum* subsp. *suis*, *B. bifidum*, and *Bifidobacterium kashiwanohense* [94]. The high growth of most strains of *Bifidobacterium breve*, *B. longum* subsp. *infantis*, and *Bifidobacterium pseudocatenulatum* on 2′-FL, 3′-FL, and difucosyllactose (DFL) is mediated by their transport into the cell via the substrate-binding protein SBP and the ABC transporter [95]. The absence of 2′-FL and LNFP I from the milk of non-secretory mothers leads to a delay in *Bifidobacterium* spp. colonization of the intestine in newborns [96].

However, Fuc release from HMOs by different *Lactobacillus* and *Bifidobacterium* species and strains [71,92,97] does not always mean the ability to metabolize it [97]. *B. longum* subsp. *infantis* strain DSM 20088 and TPY12-1 and *B. longum* subsp. *suis* strain BSM11-5 metabolize Fuc to 1,2-propanediol, whereas *B. bifidum* and *B. kashiwanohense* excrete it into the supernatant. They metabolize Fuc via non-phosphorylated intermediates to L-lactate and pyruvate via the enzymes Fuc mutarotase, Fuc dehydrogenase, L-fuconolactone hydrolase, L-fuconate dehydratase, L-2-keto-3-deoxyfuconate hydrolase, and L-2,4-diketo-3-deoxy-fuconate hydrolase [94]. The *B. longum* SC596 gene cluster for the utilization of fucosylated HMOs encodes proteins for their import, Fuc metabolism (L-fuconate dehydrotase, L-fuconate dehydrogenase, dihydropicolinate synthase, Fuc mutarotase), and two α-L-fucosidases [98].

Although the genes of *Lactobacillus acidophilus* NCFM [99], *Lactobacillus johnsonii* NCC 533 [100], and *Lactobacillus gasseri* ATCC 33323 [101] encode many catabolic carbohydrate pathways, they do not have sialidases, endo-β-1,3-*N*-acetylglucosaminidase, or α-L-fucosidases. The genomes of the *Lactobacillus casei*/*Lactobacillus paracasei*/*L. rhamnosus* group have a high percentage (up to 67%) of the main orthologous groups. Up to 23% of genes in the pangenome and 34% in the variome of *L. rhamnosus* encode functions related to carbohydrate transport and metabolism [102]. Some intestinal isolates of this group (*L. casei* LC2W, BD-II, BL23; *L. rhamnosus* GG and HN001) show the presence of genes encoding α-L-fucosidases [103]. The low homology of the three α-L-fucosidase genes from the GH29 family in *L. casei* BL23 (21% identity) determines their different substrate specificities for fucosylated HMOs [104]. Five α-L-fucosidase genes from strains LC2W and BD-II have 100% identity with the gene sequence from strain BL23, while identity in *L. rhamnosus* strains is 75–95% [103]. Six of 11 tested *L. casei* strains and two of six tested *L. rhamnosus* strains could ferment Fuc-α1,3-GlcNAc due to α-L-fucosidase homologous to AlfB, while *L. casei* BL87 and *L. rhamnosus* BL327 can also ferment Fuc-α1,6-GlcNAc [97]. When grown on Fuc-α1,3-GlcNAc, *L. casei* BL23 does not metabolize Fuc [103]; however, Fuc is critical for substrate transport into the cell through AlfH in the case its growth on Fuc-α1,6-*N*-GlcNAc-Asn [92].

Fuc metabolism gene clusters have been found in some isolates of *L. rhamnosus* (strain GG, HN001, LRHMDP2, LRHMDP3), *L. casei* ATCC 393, and *Lactobacillus zeae* ATCC 15820 [105]. The Fuc metabolism has been shown for *L. rhamnosus* strains BL358, BL377, and GG [97,105]. *L. rhamnosus* GG metabolizes Fuc to produce 1,2-propanediol under anaerobic growth conditions or lactate under aerobic conditions, apparently relying on the fucosidase activity of other intestinal bacteria, as its own α-L-fucosidases are intracellular proteins [105]. *L. zeae* ATCC 15820 can ferment Fuc, in contrast to *L. casei* ATCC 393, which has a frame shift in the *fucK* gene. Ungrouped homologues of some *fuc* genes have been found only in the genomes of *Lactobacillus shenzhenensis* LY-73T, *Lactobacillus composti* JCM14202, and *Lactobacillus herbinensis* DSM16991; therefore, it is believed that the *fuc* operon is exclusive for *L. rhamnosus* and the *L. casei*/*L. zeae* group. The *fuc* operon genes were not found in some sequenced *L. paracasei* strains [105]. Nevertheless, the production of propionate by *L. paracasei* CNCM I-3689, but not *L. rhamnosus* CNCM I-3690, has been reported in antibiotic-induced microbiota dysbiosis in mice. This led to decreased content of vancomycin-resistant enterococci and the restoration of representatives of Bacteroidetes [106]. In humans, *L. rhamnosus* GG supplementation also resulted in decreased levels of vancomycin-resistant enterococci [107,108].

## 7. Lactobacilli and Bifidobacteria Cell Wall Components and Adhesins and Immune Response Modulation

Bifidobacteria and lactobacilli use various adhesive proteins, which are often glycosylated and can modulate the immune response. Part of bifidobacteria (e.g., some strains of *B. adolescentis*, *B. longum*, *B. breve*, *B. catenulatum*, *B. pseudocatenulatum*) [109] and lactobacilli (e.g., some strains of *L. acidophilus*, *L. helveticus*, *L. crispatus*, *L. amylovorus*, *L. gallinarum*, *L. gasseri*, *L. johnsonii*) [110] can form a protein layer (S-layer protein, Slp) on the cell surface [109,111]. *slp* genes are expressed constitutively. Some lactobacilli strains do not form Slp (e.g., *L. delbrueckii* subsp. *bulgaricus* and some *L. casei* [110]). Several cell surface adhesive proteins are multifunctional; these include transaldolase, glutamine synthetase, enolase, bile salt hydrolase, phosphoglycerate mutase [112], glyceraldehyde-3-phosphate dehydrogenase, the elongation factor Tu, and the chaperonin GroEL [113,114]. Further, fibronectin-binding proteins (Fbp) [115] and other adhesive Slp have been discovered. The adhesive Slp may determine the different regiospecific colonization abilities of bacteria in the GI tract [113,114].

However, pili are the main adhesion molecules [111,116]. Gene clusters encoding pili have been found in the *B. bifidum* PRL2010 genome (three clusters of sortase-dependent *pil*, for which expression was shown for *pil2* and *pil3*) and *B. breve* UCC2003 (Type IVb [Tad] tight adhesion gene cluster) [117]. *L. paracasei* subsp. *paracasei* has three putative clusters of sortase-dependent pili that have been expressed *in vitro*. Although mass spectrometric analysis did not confirm their presence, it found surface adhesins such as Tu and GroEL. *L. paracasei* whole cells are adhesive to α-2,3-Sia, α-1,2-Fuc-containing glycans including blood group/histocompatibility antigens A, B, and O, and Le^x^, Le^y^, and Le^b^ [114]. Pili are also involved in adhesion to milk glycoconjugates, as shown for *L. rhamnosus* GG, which binds β-lactoglobulin via the pili SpaCBA. Their interactions with milk proteins are regulated by the surface composition of bacteria [116].

*Lactobacillus* and *Bifidobacterium* can synthesize EPS, which differs in terms of physicochemical properties and molecular weight depending on the source of carbohydrates [74] and the growth conditions of bacteria. The synthesis of two structurally different EPS allows *B. breve* UCC2003 to adapt to gut conditions (bile acids and pH) and avoid immune responses by masking surface antigens. The adjacent but oppositely oriented *eps1* and *eps2* operons are responsible for EPS synthesis. They have a single promoter in the intergenic region that triggers the transcription of *eps1* only, but can reverse its orientation through a mechanism that likely involves site-specific DNA inversion, which allows *eps2* transcription [118]. Lactobacilli can synthesize both homo- and heterosaccharide EPS [119]. EPS of human gut isolates of lactobacilli (various strains of *L. casei*, *L. rhamnosus*, *L. plantarum*, *L. vaginalis*) and bifidobacteria (*B. bifidum*, *B. breve*, *B. animalis*, *B. catenulatum*, *B. longum* subsp. *longum*, *B. longum* subsp. *infantis*, *B. longum*, *B. pseudocatenulatum*) often contain Glc, Gal, and rhamnose (Ram) [120,121,122,123]. The presence of Fuc in their EPS, however, has not been reported.

The interaction of various cell surface components of some lactobacilli and bifidobacteria with immune cells may contribute to an anti-inflammatory response. Several lactobacilli promote Treg induction through the creation of a Treg-friendly environment [124]. *L. rhamnosus* Lr32 and *Lactobacillus salivarius* Ls33 induce DC differentiation into tolerogenic DCs and high IL-10 production by human peripheral blood mononuclear cells, but not by mouse bone marrow-derived DCs, in which high IL-10 production is induced by *L. acidophilus* NCFM and *Lactococcus lactis* MG1363 [125]. *L. rhamnosus* JB-1 initiates the induction of Foxp3^+^ Treg cells via binding to DC-SIGN and TLR2 activation on DCs, leading to IL-10 secretion and IL-12p70 suppression [27]. *L. rhamnosus* GG colonization of 2-week-old mice induced IL-10Rβ mRNA expression but not IL-10 mRNA expression. IL-10R activation triggered STAT3 phosphorylation, which increased the colonic expression of SOCS-3 and attenuated the production of the pro-inflammatory cytokines MIP-2 and TNF-α [126]. In *Salmonella*-infected mice, *L. rhamnosus* GG increased IL-10 levels and decreased myeloperoxidase levels [127]. Cytokine production by human DCs depends on the glycan type on SpaCBA from *L. rhamnosus* GG, which can contain the Man and Fuc residues recognized by DC-SIGN. Man-containing structures enhance the expression of TLR2-induced pro-inflammatory cytokines, while Fuc-containing ligands increase IL-10 production and inhibit IL-6 and IL-12 expression [128]. The interaction of SpaCBA with murine RAW 264.7 macrophages induced IL-10 mRNA expression and decreased IL-6 mRNA expression in the macrophages [129].

Comparison of the ability of 27 lactobacilli strains and 16 bifidobacteria strains to stimulate bone marrow DCs [130] showed that most lactobacilli strains, including *L. acidophilus*, *L. gasseri*, *L. casei*, and *L. plantarum*, induced strong IL-12 and TNF-α production and the upregulation of maturation markers. In contrast, all *B. bifidum*, *B. breve*, *B. longum* subsp. *infantis*, *B. animalis* subsp. *lactis*, and some lactobacilli strains were weak inducers of IL-12 and TNF-α. IL-10 and IL-6 levels showed less change and no correlation with IL-12 and TNF-α. DCs stimulated by potent IL-12–inducing strains also produced high levels of IFN-β. When the two strains were combined, low IL-12 inducers inhibited IFN-β production, as well as the cytokine IL-12 and Th1 skewing. The immunostimulatory strains *L. acidophilus*, *L. gasseri*, *L. paracasei/casei*, and *L. plantarum/paraplantarum* induced IFN-β and IL-12 production by DCs, while some strains of *Lactobacillus reuteri*, *L. rhamnosus*, *L. paracasei*, and *L. paraplantarum* inhibited IFN-β/IL-12 induction. IFN-β induction was mediated through JNK independently of the stimulating strain, but the inhibitory bacteria suppressed the JNK effect by inducing higher levels of the transcription factor c-Jun dimerization protein 2 [130]. *B. breve* strain Yakult, but not *L. casei* strain Shirota, activated intestinal CD103^+^ DCs in mice to produce IL-10 and IL-27 via the TLR2–MYD88 pathway, which induced T cell differentiation into IL-10–producing Treg type 1 (Tr1) cells in the colon [131]. High Slp A (SlpA) expression in *L. acidophilus* L92 correlated with high induction of IL-12p70 secretion during splenocyte stimulation in vitro [132]. The CmbA and MUB adhesins of *L. reuteri* strains ATCC PTA 6475 and ATCC 53608 interacted with human moDCs and could elicit moDC-mediated Th1 and Th17 immune responses. MUB activated moDCs and induced Th1-polarized immune responses associated with increased IFN-γ production by binding to DC-SIGN or dectin-2 [133].

*B. longum* subsp. *infantis* 35624 promoted the induction of Foxp3^+^ Treg cells in human peripheral blood through TLR2-, DC-SIGN-, and retinoic acid–dependent activation of myeloid DCs and indoleamine 2,3-dioxygenase (IDO)-dependent activation of plasmacytoid DCs [28]. Its EPS, containing a branched repeat unit from 2Gal: 2Glc: 1GalA: 1 6-deoxy-L-talose [134], suppressed the host pro-inflammatory and local Th17 response in mice [135]. The percentage of sugars in *L. reuteri* Mh-001 EPS when Gal > Ram > Glc contributed to the anti-inflammatory activity of EPS-treated murine macrophages [123]. It is believed that the immune response is influenced not only by the composition and ratio of monosaccharides, but also by molecular weight (high–molecular weight EPS most often leads to suppression of the immune response) and charge (acidic EPS carrying PO_4_^-^ are strong stimulants, while neutral ones do not affect the immune response) [121].

## 8. Bacteroid Cell Wall

The successful colonization of the mouse intestine by *Bacteroides* is ensured by the incorporation of exogenous Fuc into the glycan capsule due to the presence of the bifunctional fucokinase/Fuc-1-P-guanylyltransferase (Fkp) [136]. This is believed to allow *Bacteroides* to avoid elimination by the host’s innate immune system during colonization of the intestine [137]. *Bacteroides* glycoproteins are still poorly investigated, but they contain Fuc recognized by *Aleuria aurantia* lectin and show phase variability [138,139]. There is an inverse correlation between the synthesis of *B. thetaiotaomicron* glycoproteins and polysaccharides [140]. The involvement of glycoproteins in biofilm formation [141] may contribute to the retention of *Bacteroides* in the intestine until their density is high. However, it is not known whether Fuc-containing glycoproteins are involved in biofilm formation. Part of the *Bacteroides distasonis* (reclassified as *Parabacteroides distasonis*) glycoproteins is included in the S-layer that is absent from *B. thetaiotaomicron* and *B. fragilis* [138].

*Bacteroidescaccae*, *Bacteroides ovatus*, *B. thetaiotaomicron*, *B. uniformis*, *Bacteroides vulgatus*, and *B. fragilis* [139] share a common *O*-glycosylation system, where glycan is added to the Ser and Thr residues in the D (S, T) (A, L, V, I, M, T) motif [142].

Investigated the most often, *B. fragilis* NCTC9343 can synthesize eight polysaccharides (PSA–H) and change its capsule composition based on the environmental condition. The expression of seven of the polysaccharides is controlled by a site-specific recombinase: multiple promoter invertase (Mpi), which inverts the promoter regions for phase variation. The control of PSC expression is independent of that of the other polysaccharides [143]. PSB contains terminal α1,2-Fuc [144], and PSC, PSD, and PSH are bound by *A. aurantia* lectin that is specific to α1,3-Fuc and α1,6-Fuc [136]. Based on these data and the presence of α1,3-fucosyltransferase in this *B. fragilis* strain [35], we may assume that a structure corresponding to Le^x^ antigen could be present in PSC and/or PSD, and PSH. PSC and PSB are important for colonization of the intestine, as double deletion of their genes leads to the loss of colonization resistance of the mutant due to decreased IgA-mediated adhesion. Moreover, PSB synthesis can be compensatory in PSC gene deletion [145]. In addition, PSA, PSB, and PSC have hemagglutinating properties, which correlate with the colonization ability of *B. fragilis*. PSA causes lower hemagglutination, while PSC causes the highest, and PSC and PSB together can compensate for the absence of PSA in hemagglutination reactions. The highest hemagglutination has been observed for blood group antigen O, and less for the A and B groups. PSC expression is believed to be an independent PSB duplicating system for colonization and hemagglutination [146]. PSA is non-covalently bound with PSB in the capsule [144].

The structure and composition of capsular polysaccharides differ even between strains, as has been shown for the zwitterionic *B. fragilis* 638R PS A2 and *B. fragilis* NCTC9343 PSA. In addition, PS A2 contains α1,2-Fuc, unlike PSA [144,147]. PSA is important not only in gut colonization, but also in immune system maturation [144]. During *B. fragilis* colonization of germ-free mice, PSA corrected systemic T cell deficiency and Th1/Th2 imbalance, directing lymphoid organogenesis [148]. PSA presentation by plasmacytoid DCs affected the programming and maturation of thymic progenitor cells in germ-free neonatal mice [149]. Isolated PSA interacts with different receptors, apparently depending on the presence of the lipid section. The pure polysaccharide binds to DC-SIGN on human CD11c^+^ DCs [150]. The *B. fragilis* LPS signal goes through TLR2 to the plasmacytoid DCs [151,152]. The presence of a PSA-bound lipid structure triggers signaling when interacting with TLR2/1 and dectin-1 on plasmacytoid DCs via the PI3K signaling pathway. This induced IL-10 production by CD4^+^ T cells when they were co-cultured with plasmacytoid DCs in vitro [153]. Several studies have reported that PSA promotes the induction of Treg cells in mice and IL-10 production including by human Treg cells in vitro [152,154,155]. Unlike the isolated PSB, PSA demonstrated a protective effect in induced experimental colitis [154]. The effects of Fuc-containing polysaccharides of *B. thetaiotaomicron* ATCC 29741, *B. vulgatus* ATCC 8482, *B. ovatus* ATCC 8483, *B. uniformis* ATCC 8492, and *B. distasonis* ATCC 8503 (reclassified as *P. distasonis* ATCC 8503) [136,139] on the immune response have not been studied.

The synthesis of the eight capsular polysaccharides (CPS) of *B. thetaiotaomicron* VPI-5482 is also controlled through phase variation [156] depending on host diet, the availability of certain mucin *O*-glycans, and environmental factors [157,158]. This provides competitive fitness for bacteria in the gut and allows them to evade the host’s immune response [158,159]. However, which polysaccharides contain Fuc is unknown. CPS4 contains a species-specific immunogenic epitope 225.4 [160]. CPS4 expression decreases when HMOs are utilized [161], is suppressed during biofilm formation, and increases when the infant switches from breastfeeding to solid foods [140]. CPS1, CPS2, CPS3, CPS5, and CPS6, as well as CPS4, inhibit biofilm formation, while CPS8 (and potentially CPS7) exhibits adhesive properties, and its locus has homology with FimA, the main component of type V pilus [141].

The glycolytic potential of *B. uniformis* IECT 7771 is greater than that of *B. thetaiotaomicron* [162], while *B. vulgatus* and *B. fragilis*, defined as pathobionts [163], have the smallest set of genes for glycan degradation. During *B. uniformis* IECT 7771 growth on pectin, the genes involved in polysaccharide synthesis, including Fuc2NAc synthesis, are activated, while mucin glycans induce the highest expression of genes for proteins involved in butyrate production [162]. Bacterial polysaccharides can also be a source of fermentable glycans in the intestine [64]. The addition of *Bifidobacterium* EPS to *B. fragilis* growth medium increased propionate production regardless of the type of medium (minimal or rich), although the production of both propionate and butyrate was higher in the rich medium than in the minimal medium [164].

## 9. Helicobacter Pylori LPS

*H. pylori* is a member of the human gastric microbiota and is often regarded as a pathogen. It colonizes almost half of the world’s population, but only 10–20% cause several diseases, including stomach cancer [165]. Nevertheless, there is every reason to consider *H. pylori* a pathobiont that co-evolved with humans and whose early colonization has positive effects on the host, such as the regulation of the hormones leptin and grainin [166], and protection against allergic and autoimmune diseases, and IBD [167,168]. The LPSs of different *H. pylori* isolates can carry structures similar to type 1 H antigen (but not type 2 H), Le antigens (Le^a^, Le^b^, Le^x^, Le^y^, sialyl Le^x^), blood group antigens A and B in some strains, and polylactoseamine (i-antigen) [165]. LPS synthesis is also characterized by phase variations [169]. These antigen phase variations are often called spontaneous, but most likely, they also depend on the presence of nutrients and environmental conditions in the GI ecosystem.

DC-SIGN recognition of the Le^x^ and Le^y^ monoantigens in *H. pylori* promotes the development of a tolerogenic Th2 response, while their absence induces a strong Th1 response. The *H. pylori* poly-Le^x^ antigen is recognized by the galectin-3 receptor [170]. The immune system responds to *H. pylori* heterogeneous population with Th1/Th2 responses, which generally maintains the Th0 response [169]. Successful *H. pylori* colonization is largely due to its ability to produce Le antigens of similar type to the host Le antigens [171,172]. The absence of Le antigens in LPS results in a significant decrease in the ability of *H. pylori* to induce TNF-α secretion compared to LPS containing Le^x^/Le^y^ or standard *Escherichia coli* LPS [172]. The Th2 response is considered a host tolerant response to a potentially damaging factor when its benefits for the host exceed the risks of its presence. It is likely that *H. pylori* colonization of newborns with the corresponding genotype is facilitated by Th2 immunity and subsequently leads to the development of immune tolerance. At the same time, chronic gastritis is accompanied by Treg cell development, and its severity leads to a shift in the balance towards Th17 cells, which indicates the presence of another control by the immune system [173]. Th17 cells induce IL-23 production by DCs while limiting *H. pylori* growth but increasing disease severity [174]. The Th1 response is most likely determined by both types of LPS and the time of colonization, which provokes the acute course of the disease [175]. The main adhesin of *H. pylori* BabA mediates its binding to gastric ECs via Le^b^ and type 1 H antigens expressed on healthy gastric mucosa, producing autoantibodies to Le antigen LPS. SabA binds to sialylated carbohydrates, mediating adhesion to the inflamed gastric mucosa [176].

## 10. Fucosylation of IECs

The fucosylation of glycoconjugates of epithelial and goblet cells of the stomach, distal ileum, and large intestine of the host is necessary for establishing symbiotic relationships with microorganisms. This process is integrative, as it is controlled by both bacteria and the host, allowing not only the maintenance of symbiotic relationships, but also the avoidance of excessive bacterial load [177,178,179]. FUT2 is responsible for α1,2-fucosylation of EC membrane glycoproteins [180] and sphingolipids [181]. IEC α1,2-fucosylation is induced and varies under the influence of different factors, including stress, in contrast to M cell α1,2-fucosylation, which is responsible for constitutively expressed FUT1 [182]. In addition to maintaining symbiotic microbial relationships with the host, fucosylation enhances colonization resistance to some pathogens (e.g., *Citrobacter rodentium* [183], *Salmonella typhimurium* [184], *Enterococcus faecalis* [185]). However, in dysbiosis, pathobionts and pathogens can gain an advantage for growth [186,187,188].

Experiments in mice and rats have shown that fucosylation occurs in two stages. The first stage is the period of intestinal maturation during breastfeeding, which is regulated by glucocorticoid hormones (at the transcriptional level) and nutrients, including polyamines [180,181,189]. In mice, spermine and spermidine induce the synthesis of α-1,2-fucosylated, but not sialylated, glycoproteins, increasing FUT2 and α-L-fucosidase activity [180]. The source of polyamines for infants in the postnatal period is breast milk, which contains comparable amounts of spermine and spermidine, and a minor amount of putrescine. Their content in breast milk can vary depending on the maternal diet and milk maturity. Further, both plant and animal products and bacterial metabolites, mainly Enterobacteriaceae and *Clostridium* spp., are a source of polyamines. Oral administration of polyamines in the postnatal period improves the maturation of intestinal immune cells and increases IgA levels in intestinal villi and crypts [190].

The second stage, the period of intense fucosylation, begins with the transition to solid food. Here, the main role belongs to bacterial factors, in particular representatives of *Bacteroides* [177,189,191] and segmented filamentous bacteria (SFB) [184,192]. During this period until the end of intestinal maturation, insulin levels are correlated with increased α1,2-fucosyltransferase activity [193], but sensitivity to glucocorticoid hormones disappears, apparently due to a decrease in the number of cytoplasmic hormone receptors [180]. The hormone action periods differ slightly between rats and mice, possibly due to the difference in gut maturation. In addition to these hormones, regulation by thyroxine [180] is assumed, but is poorly investigated.

Induction by *B. thetaiotaomicron* α1,2-fucosylation of ECs in the distal small intestine of the host is closely related to Fuc metabolism in bacteria, which is controlled by Fuc levels in medium and in the cell [191]. *Bacteroides* mutants unable to metabolize Fuc cannot do this [177,189].

*Bacteroides* are detected in the first week of an infant’s life; they enter the intestine mainly during passage through the birth canal [194,195], as they are not always present in milk [78]. *B. vulgatus*, followed by *B. uniformis*, and then *B. fragilis*/*B. thetaiotaomicron* are found most frequently in breastfed infants at day 7 of life. By the end of 1 month of life, the prevalence of *B. vulgatus* falls, while the prevalence of other *Bacteroides* increases significantly [196].

Strains of *B. thetaiotaomicron*, *B. fragilis*, *B. caccae*, *B. ovatus*, and *B. stercoris* can metabolize both HMOs and mucins with different substrate specificities [72,161]. *B. thetaiotaomicron* VPI-5482 (ATCC 29148) expresses genes for the fucose utilization pathway (Fuc isomerase, L-fuculose kinase, L-fuculose-1-phosphate aldolase, lactaldehyde reductase) to L-lactate aldehyde and dihydroxyacetone phosphate. This pathway is controlled by FucR, which binds Fuc at high cellular levels and activates the transcription of genes of this pathway, including the *fucR* gene. At the same time, FucR represses the transcription of another genetic locus responsible for producing a bacterial signal that directly or indirectly triggers the synthesis of IEC fucosylated glycans [197]. Neither the genetic locus nor the produced bacterial signal has been identified. Switching to polysaccharides from solid food enhances the growth of *Bacteroides*. When *B. thetaiotaomicron* has reached a certain density in the intestine, the level of Fuc available to it decreases [191]. The decreased Fuc cellular levels leads to the repression of its catabolism and to the activation of the IEC initiation fucosylation signal [197]. Further studies suggest that the initiation signal may be butyrate, which is obtained as a result of processes occurring during changes of the gut bacterial composition. However, its concentration must reach a certain critical value to initiate α1,2-fucosylation. Experiments with fecal suspensions have shown that the conversion of lactate to propionate or butyrate, and their ratio, are influenced by the medium pH and the presence of polysaccharides. In the presence of polysaccharides, lactate is predominantly converted to acetate and butyrate, and in its absence, mostly to propionate, especially at an initial pH of 6.4 [198]. *Bacteroides* have butyrate and propionate production pathways [199], and when switching from HMOs to polysaccharides, the production of these SCFAs increases significantly. So, the gut conditions can support the growth of *Bacteroides* on the exogenous polysaccharides of complementary feeding and increased butyrate production. Besides, the role of butyrate in the initiation of fucosylation confirms the study of the effect of certain food and microbial metabolites on the synthesis of complex and hybrid membrane *N*-glycans of differentiated Caco-2 and partially differentiated HT-29 IECs [200]. Lactate, acetate, and butyrate stimulated *N*-glycan fucosylation in Caco-2 cells by 41.6%, 60%, and 72%, respectively, and in HT-29 cells by 5%, 35%, and 262%, respectively, while the increased *N*-glycan fucosylation was accompanied by a decrease in their sialylation [200].

The bacterial symbionts acting through a certain receptor on IECs stimulate the intracellular signaling pathways ERK and JNK, as a result of which the nuclear transcription factors c-Jun and ATF2 are activated. This induces *fut2* mRNA transcription and leads to increased FUT2 activity in the Golgi, followed by increased expression of fucosylated glycans on the colon mucosal cell surface [201]. Studies on the SFB-initiated fucosylation mechanism helped to establish this receptor.

SFB colonize the distal ileum. They belong to Clostridiales, as more than 70% of their genome is homologous to that of *Clostridium* spp., while the remainder has homology with *Bacillus*, *Thermoanaerobacter*, and *Ruminococcus* species. Due to their reduced genome, SFB are highly dependent on the host for essential nutrients, and as a result, are specific to it [202]. Human and rodent SFB belong to different clades. SFB have genes for glycan cleaving and importing its fragments, and for metabolizing GlcNAc, Man, and sialic acids, but not Fuc, Gal, or GalNAc. The presence of sialidase and fucosidase genes in mouse SFB has not been reported [202]. Interestingly, human SFB have a sequence [203] encoding β-glucosidase (EC 3.2.1.21), with possible β-D-fucosidase activity for hydrolyzing terminal unreduced β-D-Fuc residues in β-D-fucosides.

During weaning, SFB start to grow actively after the disappearance of breast milk sIgA, which suppresses their growth, and limits access to IECs [204] until the infant develops its own antibodies to them. SFB stimulate the formation of lymphoid follicles and tertiary lymphoid tissues in the host, which leads to increased sIgA concentrations and the number and activity of IgA-secreting B cells [204]. SFB can attach to IECs without damaging them, but with induction of the expression of their *nos2*, *duoX2*, *duoXA2*, *saa3*, *tat*, and *lcn2* genes [205] and rearrangement of the IEC cytoskeleton at the contact site [202]. SFB induce the appearance of Th17 cells producing IL-17 and IL-22 [205,206]. T cell priming and their differentiation into Th17 cells occurs locally in the lamina propria [207] after the interaction of SFB flagellin with TLR5 CD11c^hi^CD11b^hi^ DCs. TLR5 recognizes highly conserved motifs in SFB flagellin, which are almost absent in other *Clostridium* species [204]. Apart from Th17 cells, SFB can induce Th1 and possibly Tfh cells [208].

SFB, but not *Lactobacillus murinus* [184], and LPS pathogens acting on TLR4 of small intestine ECs during acute infection [183] can significantly increase α1,2-fucosylation. In these cases, fucosylation is controlled by group 3 innate lymphoid cells (ILC3). CD90^+^RORγt^+^ ILC3 from the lamina propria promotes the development and maintenance of secondary lymphoid tissues through gut microbiota–independent lymphotoxin expression. The continuous production of lymphotoxins contributes to the induction of FUT2 [184]. However, fucosylation is significantly increased in response to IL-22 production by ILC3 under the influence of IL-23 produced by DCs. IL-22 binds to IL-22 receptor (IL-22R), which is expressed by IECs, resulting in the induction of Fut2 [183]. IL-22R is a heterodimeric complex of IL-22RA1 and IL-10Rβ. IL-22 has high affinity for IL-22RA1, binding to which increases the affinity for IL-10Rβ [209].

Returning to the unknown bacterial factor that acts as the receptor of IECs, we may assume that this receptor is IL-22R, as IL-22 and IL-22R participation in ERK and JNK signaling pathway activation has been demonstrated [210,211]. SCFA production from polysaccharides regulates the ILC3 response through GPR43 [212]. Propionate induces the expansion of CCR6^+^ ILC3 expressing free fatty acid receptor 2 (Ffar2, also known as GPR43) and increases their IL-22 production through the AKT and STAT3 signaling pathways [213]. Butyrate increases IL-23 production by DCs in a Stat3-independent manner [214].

Fucosylation is also controlled by IL-10–producing CD4^+^ T cells expressing αβTCR [215]. In germ-free mice, the number of cells with αβTCR increased 10-fold 1 month after the initiation of colonization and was five times higher than that of cells expressing γδTCR [192]. IL-10 plays a key role in downregulating epithelial fucosylation in the intestine by regulating Fut2 expression. Non-fucosylated IECs express only one IL-10 receptor (IL-10R) subunit, while fucosylated IECs express both subunits, i.e., α and β. IL-10 acts directly on IL-10R on fucosylated IECs. It inhibits the p38/MAPK pathway that activates ATF2. A similar mechanism may be responsible for the IL-10–mediated inhibition of Fut2 expression and the associated fucosylation of IECs. IL-10 is required but is not sufficient for downregulating epithelial fucosylation, requiring additional molecules on the surface of CD4^+^ T cells and/or produced by these cells [215]. The IL-10Rα subunit is ligand-binding and unique to IL-10. The IL-10Rβ subunit is common to other type II cytokine receptors, including receptors of IL-22, IL-26, and IFN-λ, and is required for activating the downstream kinases [216]. Butyrate induces IL-10Rα subunit expression through Stat3 activation and HDAC inhibition. It also enhances the barrier function of human IECs and represses permeability-promoting claudin-2 tight junction protein expression through an IL-10Rα–dependent mechanism [217].

## 11. Intestinal Immunoglobulins

A subset of B cells called B1 cells develops from progenitors in the omentum or liver of the fetus. According to availability of the CD5 marker, besides the combined markers CD20^+^CD27^+^CD43^+^CD70^−^, B1 cells are divided into two subpopulations. The CD5^+^ B1a subpopulation is usually absent from the bone marrow in adults, while CD5^-^ B1b cells are present not only in the fetus, but also in the bone marrow of adult humans, ensuring the maintenance of the pool of B1 cells. The subset of B cells absent from the fetal omentum are B2 cells. B2 and B1b cells are the predominant progenitors of immunoglobulin-producing plasma cells [218]. B1a cells are the main producers of the so-called natural antibodies, which, despite their microbiota-reactive specificity, can arise in the absence of exogenous microbiota and are represented mainly by antibodies of the IgM class, as well as IgA and IgG [219]. Antibodies to Galβ1,3GlcNAc (Le^c^), 4-HSO_3_Galβ1,4GalNAc, Fucα1,3GlcNAc, Fucα1,4GlcNAc, GalNAcα1,3Gal, Galα1,4Galβ1,4Glc, Galα1,4Galβ1,4GlcNAc, GlcNAcα-terminating glycans, LNnT and lacto-*N*-tetraose (LNT), and a variety of other carbohydrate structures, including the ABO blood group epitopes, have been identified. At the same time, natural antibodies to Le^x^ and Le^y^ antigens are not observed in healthy people [220]. The content of natural antibodies is typically low; they are characterized by low binding affinity, and their production requires TLR activation of B cells independent of T cells. The function of natural antibodies is not known. By the eighth month of an infant’s life, the appearance of antibodies produced by B2 cells recognizing ABO and α-Gal antigens in addition to natural IgM indicates the maturation of specific antibodies to these antigens [218].

Unlike mice, humans have two IgA subclasses: IgA1 and IgA2, which can exist in different polymeric forms. They differ in their specificity for human moDC receptors: IgA1s preferentially bind to FcαRI and CD71, while IgA2s bind to dectin-1 and DC-SIGN, and their affinity for receptors increases upon dimerization [221]. Dectin-1 binds sialylated glycan residues of IgA2 together with the Siglec-5 receptor [222].

Colonization of the intestine in newborns triggers sIgA production. sIgAs are mainly induced in gut-associated lymphoid tissue (Peyer’s patches in the small intestine, mesenteric lymph nodes [germinal centers are present in both types of tissue], isolated lymphoid follicles, and blind spots), which contains up to 80% of plasma cells and 90% of the body’s sIgA [223]. In the germinal centers, IgA-producing plasma cells differentiate both by T cell–dependent (TD, B cell activation by CD4^+^ T cells through the interaction of CD40–CD40L ligands and TGF-β secretion required for IgA production), and by T cell–independent (TI, CD4^+^ T cell–independent activation of IgA production) pathways [219,223]. In the former, this leads to the formation of high-affinity, antigen-specific IgA1s, which bind to antigens in a canonical manner and neutralize pathogens, or selectively regulate the physiology of some commensal bacteria. IgA1s can show specificity not only for proteins, but also for glycans exposed on the intestinal bacteria surface (O antigens, polysaccharide capsules, flagellar antigens, teichoic acids). In TI pathways, polyreactive, low-affinity IgA2s are produced, which have wide reactivity to diverse, but nevertheless, specific subgroups of microbiota [224], most likely due to the specificity of bacterial adhesins to IgA glycans and the understudied glycan–glycan interactions [225]. This non-canonical binding of IgA2 to symbiotic bacteria contributes to the maintenance of colonic resistance of bacteria in the intestine [223,226,227]. sIgA of both subclasses have different tissue distribution: Peyer’s patches contain more IgA1^+^ B cells than lamina propria, and the colon contains mainly IgA2^+^ B cells [227,228]. As both subclasses are actively involved in maintaining intestinal homeostasis, they are also termed homeostatic IgA [229].

In contrast to monomeric IgAs in serum, sIgAs are polymers, more often dimers, linked by a J chain and a polymeric Ig receptor (pIgR) secreted in the mucosal fluid. Human pIgR is a transmembrane protein whose extracellular domain, also called the secretory component (SC), is composed of five Ig domains. pIgRs are also expressed during breastfeeding in the mammary glands and in the liver in adults, which facilitates the transport of sIgA to milk and bile, respectively [227]. The heavy (H)-chain sIgA and SC are highly glycosylated. On the one hand, glycans protect sIgAs from proteolysis in the intestine [219] and are a carbon source for bacteria [230]. On the other hand, their glycans include Fuc, Man, Gal, and sialic acids [231] to allow the binding of a wide range of bacteria in comparison with other classes of specific antibodies due to their carbohydrate composition [219], as they act as traps for bacterial adhesins such as milk oligosaccharides or mucins. Glycosylation has mainly been studied with colostrum sIgA. *N*- and *O*-glycosylation of sIgA1 and sIgA2 are different. sIgA1 has an additional 23–amino acid sequence in the hinge region with multiple *O*-glycosylation sites, allowing the Fab fragment to maintain a specific conformation. *N*-glycans on SC and *O*-glycans in the hinge region H chain of sIgA1 represent a wide range of glycan epitopes, including structures with Le and sialylated Le antigens, and with 1,2-Fuc. Potentially, these epitopes can bind to different bacterial adhesins, such as that shown, for example, for *H. pylori*, *E. coli*, *Clostridioides* (formerly, *Clostridium difficile*) toxin A, and *Streptococcus pneumoniae*. Most of the *N*-glycans on the H chains of both sIgA1 and sIgA2 have a terminal GlcNAc or Gal. The *N*-glycans on SC apparently mask the binding sites for H-chain glycans, making them available for binding after interaction with bacterial adhesins or cellular lectins. Man-binding lectin (MBL), which recognizes GlcNAc, does not bind free sIgA, unlike bound sIgA [232], suggesting that the MBL binding sites are hidden in the unbound state. Most SC *N*-glycans have a core Fuc [232,233]. Various sIgA have differing glycan structures, and their variability is highly dependent on the inflammatory process, the rate of IgA production, and antibody cloning [233]. Most likely, the structures of IgA glycans and mucins at specific time points are identical, and their recognition by both bacterial adhesins and host immune cell receptors depends on the epitopes expressed at that moment, which leads to the triggering of either tolerogenic or inflammatory responses.

The predominant intestinal sIgAs have many different functions when interacting with commensal bacteria, which depend on the time period and the intestinal region [219,223,227]. They are believed to differ functionally not only from serum IgAs, but also from the mucin layer IgAs of the lung, eyes, and urinary tract. Several functions of sIgA have been identified, including those determined by the glycans they carry: (1) the removal of pathogens with immune exclusion via non-specific immunity; (2) the development of specific immunity to them; (3) participation in the induction of tolerance to harmless dietary and commensal bacteria antigens; (4) the promotion of bacterial colonization of certain symbionts and maintaining homeostasis in the mucin layer [223], including through the regulation of bacterial gene expression and the formation of bacterial associations. The last two points form the basis for the acceptive immunity hypothesis.

## 12. Immunoglobulin Coating of Bacteria

sIgA binding to bacteria promotes host selection of symbiotic bacteria. Although most bacterial species of the gut microbiota are coated with non-specific, polyreactive sIgAs, these sIgAs have been observed in vivo to bind taxonomically distinct subgroups of the microbiota in both mice and humans [219]. The antibody coverage level of bacteria depends on breastfeeding, age, antibiotic use, and geographic location. Besides the predominant IgA, IgM and IgG are also present in the intestine. High levels of IgG and IgA in the infant’s intestine are maintained by maternal antibodies from breast milk, but gradually decrease during the first 18 months of life. IgAs and IgMs cover the same bacteria and their levels correlate with the content of various representatives of *Bifidobacteria*, and Enterobacteriaceae dominating the early microbiome OTUs, as well as with *R. gnavus*. Most Bacteroidetes, Verrucomicrobia, γ-Proteobacteria, and most Firmicutes do not show any significant association with antibodies. *Blautia* is free from antibody coating. IgG covers a smaller part of the microbiota that is significantly different from that coated by IgA and IgM. Only some representatives of Enterobacteriaceae, Erysipelotrichaceae, Veillonellaceae, and Clostridiaceae are coated with IgG [224]. Most Firmicutes in the mouse intestinal microbiota are not IgA-bound, with the exception of lactobacilli and some species of *Clostridiales* and *A. muciniphila* (phylum Verrucomicrobia) [234,235].

Analysis of the distribution of sIgA^+^ sIgM^+^ and sIgA^+^ sIgM^−^ coating bacteria revealed one more type of control of bacterial distribution in the intestine, which indicates the auxiliary role of sIgM in supporting intestinal mucosa colonization by different bacteria. Seven Lachnospiraceae and Ruminococcaceae OTUs are coated with sIgA^+^ sIgM^+^, and *Bacillus cereus*, *Roseburia*, and *Ruthenibacterium lactatiformans* demonstrate especially strong associations. IgMs bind strongly to *B. vulgatus*, but have weaker or no association with other Bacteroidetes (*B. fragilis*, *B. thetaiotaomicron*) or Proteobacteria (*E. coli*). sIgA^+^ sIgM^+^-coated bacteria are more common in the ileum, but IgM coating is not always detectable in adult humans [236].

Most commensal bacteria in the small intestine are coated with IgA1s and IgA2s, while the colon contains IgA1^+^ IgA2^+^ and IgA1^−^ IgA2^+^–coated bacteria, with IgA2s predominantly targeting Bacteroidetes. Microchip analysis of bacterial glycans showed that IgA2s almost exclusively recognize Galα-terminal glycans and predominantly sialoglycans with terminal Neu5Ac or Neu5Gc, while GlcNAc-terminal glycans bind IgA1s and IgA2s equally. Among the IgA-coated bacterial genera, IgA1^+^ IgA2^+^ coating is most often found in *Rhodococcus*, *Bifidobacterium*, and *Arthrobacter* from Actinobacteria, and IgA2^+^ IgA1^+/−^ coating in *Prevotella*, *Flavobacterium*, and *Bacteroides* from Bacteroidetes. *B. longum* binds both IgA1s and IgA2s, while *B. vulgatus* is covered only by IgA2. IgA1- and IgA2-coated bacteria increase macrophage production of IL-6 and TNF-α; however, IgA1 induces more IL-10 than IgA2 [228]. The degree of IgA coating of some microbiota representatives from different localization sites of the mouse body (small intestine, colon, mammary and salivary glands, or bone marrow) suggests that opsonization of bacteria with natural antibodies is also a mechanism for establishing homeostatic relationships in local ecosystems [234].

The stratification of bacteria, which determines their lumenal or mucosal distribution, also occurs through their binding by IgAs [145]. This probably depends on the glycan epitope type expressed on the bacterial cell surface. For example, sIgA binding to several *B. fragilis* capsular polysaccharides promotes its adhesion to mucus [145], and non-specific sIgA binding to *L. rhamnosus*, *Bifidobacterium lactis*, or non-adhesive *E. coli* increases their adhesion to Caco2 cells [237]. As the formation of capsular polysaccharides in *B. fragilis* leads to biofilm destruction, such IgA coating can be considered as an alternative mechanism for its retention in the intestinal mucin layer. The synthesis of the corresponding carbohydrate epitopes in capsular polysaccharides and LPS, flagella, and adhesins is modulated in response to changes in growth conditions and diet, so this leads to the establishment of a relationship between intestinal microorganisms, sIgAs, and diet. For example, mice on low-protein and low-fat diet lost glycan-mediated interactions between *Lactobacillus* and sIgAs due to adaptive changes in bacterial carbohydrate metabolism [238]. Increased expression of membrane and secreted proteins of *B. thetaiotaomicron*, involved in the utilization of fructan or pectin from food, leads to the production of sIgA specific to them and suppression of the locus of fructan utilization. This is considered a regulatory mechanism of microbial colonization through their metabolism modulation by the host, as on the one hand it helps to maintain the diversity of the intestinal microbiota in the event of dietary restrictions and changes in food, and on the other hand, it helps to reduce intestinal pro-inflammatory signaling [226]. *B. thetaiotaomicron* proteins BT2268 and BT2269 belong to the family of mucus-associated functional factor (MAFF); they contribute not only to colonization by *Bacteroides*, but also by Firmicutes. Monoclonal 7-6IgA binding to the BT2268 protein, a SusC homolog (TonB-dependent transporter of the starch utilization system), increases its expression as well as that of its orthologues in *B. vulgatus*, *B. fragilis*, and *P. distasonis*, thereby promoting bacterial colonization in the mucus [239]. The different affinities of sIgA to bacteria affect the elimination of some types of bacteria and eliminates the growth of others [219,229]. Variability of the binding capacity of fecal IgA to the same bacteria in different people contributes to the selection of microbiota and the formation of enterotypes, and the difference in the number of Th17, Th17.1, and Th22 cells in people with different enterotypes [240]. Therefore, the selection of taxa due to sIgA binding to the bacteria determines their effect on the host metabolism and physiology [241].

At the same time, bacteria not covered in sIgA can become pathobionts under certain conditions, and in this case, sIgAs specific to them are produced [229]. Such bacteria include SFB, *Mucispirillum* spp., *Helicobacter* spp. [229,234], an unclassified genus of the S24-7 family from Bacteroidales, unclassified Erysipelotrichaceae [234], *Streptococcus luteenzaellae*, *Haemophilus parainfluenzae*, and *Collinsella aerofaciens* [229]. It is believed that under non-homeostatic conditions, they produce surface epitopes that promote the production of specific antibodies to prevent their binding to IECs, because, for example, *Blautia* sp. under normal conditions is not coated with sIgAs [224]. Interestingly, some murine monoclonal and polyreactive antibodies recognize common epitopes of SFB and proteobacteria of the genera *Burkholderia*, *Serratia*, *Pseudomonas*, *Acinetobacter*, and *Stenotrophomonas* [219].

Besides intestinal antibodies, serum IgG and IgA targeted to intestinal bacterial glycans that recognize the glycan epitopes of both commensal and pathogenic bacteria have also been found [218]. Produced mainly by the TD pathway due to inflammation or dysbiosis, serum IgAs against intestinal bacteria trigger a stronger and more specific immune response [242].

## 13. Conclusions

As can be seen from the above, host glycan composition plays an important role in bacterial cell wall construction, the synthesis of carbohydrate patterns, and SCFA production by bacteria that support a tolerogenic immune response. Although it is believed that carbohydrate patterns can be masked in large polysaccharide sequences [9], they are most likely available for recognition by cellular lectin receptors in vivo. Both oligosaccharides and glycans of glyconjugates of milk, mucins, and immunoglobulins are involved in the immune exclusion of pathogens. They also participate in colonization by mutualistic bacteria, being a host source of food for them [70,243]. In addition, in contrast to secreted mucins, sIg is more conducive to the formation of biofilm [244] or colonies, forming a bulky structure with some bacteria through antigen–antibody complexes, which can be removed as a particle during excessive bacterial growth. It is likely that bacteria carrying carbohydrate patterns such as Le^x^ and Le^y^ are not covered with sIgA2 during colonization, and can colonize the intestines of infants without causing inflammation, as long as the bias in the direction of the Th2 response persists. The recognition of these patterns by immune cell DC-SIGN in the presentation of dietary and bacterial antigens during the training of the infant’s immune system is also important for developing immune tolerance. At the same time, bacteria can form a biofilm by attaching not only to neighboring cells through their adhesins, but can also be retained by the IECs through DC-SIGN. This can explain the lack of natural antibody coverage in some *Bacteroides* and Firmicutes, as well as *H. pylori*, in contrast to many Proteobacteria, in 6–12-week-old SPF (specific pathogen–free) mice [234]. *Bacteroides* and *H. pylori* are potentially pathobionts [163]. The transfer of maternal blood IgGs specific to other *Bacteroides* epitopes to the fetus before birth protects the infant from *Bacteroides* [245]. The blood of patients with Crohn’s disease contains increased amounts of IgGs against fucosylated glycans. This can indicate the active degradation of mucins. At the same time, many glycans have the same composition, but clearly differ structurally; therefore, the study of their structure is of interest from the viewpoint of the patterns they form [246]. The role of 1,2-Fuc–containing patterns is not clear. Most bifidobacteria and lactobacilli probably have Gal-containing patterns in their cell walls, which are recognized by sIgA, the complex with which leads to the development of a tolerogenic response. The binding of sIgA to *L. rhamnosus* CGMCC 1.3724 induces the expression of TLR regulatory proteins and RALDH2 in CD11c^+^CD11b^+^MHCII^+^ DCs from the subepithelial dome region of Peyer’s patches (but not from the spleen) and IL-10 and TGF-β production. Such sIgA coating of commensal bacteria is believed to contribute to the formation of the DC tolerogenic profile required to maintain intestinal homeostasis [247]. We believe that the contribution of Fuc as a source of propionate production in the early stages of infant immune system development is also underestimated [248]. It appears especially important given that propionate inhibits antigen-specific CD8^+^ T cell activation [69]. Regarding a number of pathogens and pathobionts from Proteobacteria (*S. typhimurium*, *Campylobacter jejuni*, *H. pylori*, *E. coli*, etc.) that have adhesins to fucosylated IECs and that can metabolize Fuc, their mechanism of action is not fully understood [249]. To conclude, the role of fucose as an important modulator of the gut microbiome and immune system with potential anti-inflammatory activity [248] should be investigated with care in future experimental and clinical studies.

## Figures and Tables

**Table 1:** Correlations between some human milk oligosaccharides and bacteria in colostrum (data from Aakko et al. [81]).

HMO	Positive Correlations, Bacteria	Negative Correlations, Bacteria
LNFP III	*Bifidobacterium breve* > *Staphylococcus aureus* > *Enterococcus* spp. > other	*Lactobacillus* group > *Faecalibacterium prausnitzii* > *Clostridium* cluster XIVa
LNFP I	*Akkermansia muciniphila* > *Bifidobacterium* spp. > *Enterococcus* spp. > *Bifidobacterium breve* > *Bifidobacterium longum* group and *Staphylococcus aureus* > *Streptococcus* group > *Staphylococcus* spp. > other	Total bacteria > *Faecalibacterium prausnitzii*
2′-FL	*Akkermansia muciniphila* > *Bifidobacterium* spp. > *Clostridium* cluster XIVa > *Lactobacillus* group	*Staphylococcus aureus* > *Enterococcus* spp. > total bacteria > *Staphylococcus* spp. > *Bacteroides*–*Prevotella* group > other
3′-FL	Total bacteria > *Lactobacillus* group > *Bifidobacterium bifidum*	*Enterococcus* spp. > *Akkermansia muciniphila* > *Staphylococcus aureus* > *Bifidobacterium longum* group > *Staphylococcus* spp. > *Bifidobacterium breve* > *Bifidobacterium* spp. > *Faecalibacterium prausnitzii* > other

HMO: human milk oligosaccharide. 2′-FL: 2′-fucosyllactose. 3′-FL: 3′-fucosyllactose. LNFP I: lacto-N-fucopentaose I. LNFP III: lacto-N-fucopentaose III. The mathematical symbol “>” refers to Spearman rank correlations between HMOs and bacterial counts.

## Data Availability

Not applicable.
